# The hidden curriculum: examining gender disparities in career trajectories of female medical graduates from Nepal

**DOI:** 10.1186/s12889-025-22700-9

**Published:** 2025-04-26

**Authors:** Animesh Ghimire, Mamata Sharma Neupane

**Affiliations:** 1 Sustainable Prosperity Initiative Nepal, Thulo Kharibot, Baneshwor-31, Bhimsengola, Kathmandu, Nepal; 2 Department of Nursing, Department of Public Health, Chitwan Medical College, Bharatpur- 5, Kailashnagar, Chitwan Nepal

**Keywords:** Gender equality, Medical education, Hidden curriculum, Female medical students, Career trajectories, Public health, Nepal

## Abstract

**Background:**

Achieving gender equality in education is crucial for promoting social equity, driving economic growth, and improving overall population health. In Nepal, deeply rooted socio-cultural norms, economic disparities, and patriarchal values greatly impact women’s access to educational opportunities, particularly in the field of medicine. Although there has been an increase in female enrollment in medical programs, pervasive biases, gendered expectations, and informal cultural scripts—often referred to as the “hidden curriculum”—continue to influence their aspirations, specialty choices, and professional journeys. Understanding these dynamics is vital for ensuring women’s full engagement in the medical workforce.

**Methods:**

This qualitative study employed semi-structured interviews with fifteen final-year female undergraduate medical students at a private medical college in Bharatpur, Nepal. Thematic analysis was employed to identify and interpret the key themes.

**Results:**

Five key themes emerged: (1) The Marriage Mandate: Negotiating Family, Tradition, and Professional Aspirations; (2) The Gendered Clinic: Unveiling Bias in Medical Education and Practice; (3) Investing in Daughters, Expecting Returns: The Gendered Economics of Medical Education; (4) Transnational Aspirations: Negotiating Mobility, Marriage, and Medical Careers and (5) Claiming Space: Agency, Resistance, and Redefining Success in the Medical Profession.

**Discussion:**

The pervasive “hidden curriculum” of gendered barriers persists despite policy efforts, constitutional safeguards, and increasing female representation in medical schools. These challenges, if unaddressed, risk perpetuating a cycle of underutilizing women’s talents, limiting the diversity of the healthcare workforce, and hindering progress toward achieving equitable health outcomes. The findings underscore the urgent need for gender-transformative approaches that acknowledge and actively dismantle these deeply rooted biases at institutional, community, and policy levels. These approaches should focus on creating supportive structures that empower women to fully contribute to the medical profession.

**Conclusion:**

Female medical graduates encounter significant obstacles, including entrenched patriarchal norms, systemic inequalities, and a pervasive “hidden curriculum” of biases. However, they demonstrate remarkable resilience and determination in challenging stereotypes, redefining success, and reimaging their professional identities. Their experiences align with global efforts toward gender parity in education and employment. Equitable representation of women in the medical workforce is not only a moral imperative but also a strategic necessity for advancing public health, strengthening healthcare systems, and promoting social justice.

**Clinical trial number:**

Not applicable.

**Supplementary Information:**

The online version contains supplementary material available at 10.1186/s12889-025-22700-9.

## Background

Gender equality in education is foundational for advancing global equity, strengthening public health, and achieving sustainable development goals. Extensive research has established that improving girls’ and women’s access to quality education yields long-term benefits for families, communities, and entire nations, enhancing their agency and economic prospects [1–3]. However, despite these recognized benefits, significant disparities persist. The United Nations Educational, Scientific and Cultural Organization (UNESCO) data from 2023 indicate that over 122 million girls worldwide remain out of school [4], representing a substantial obstacle to global progress. Investments in women’s education have been linked to increased earnings, reduced poverty, and improved health outcomes [5, 6]. For example, a World Bank study demonstrated that increasing the share of women with secondary education by just 1% point can boost annual per capita income growth [7]. Furthermore, research has shown strong correlations between maternal education and child health, with children born to mothers with secondary education experiencing significantly reduced mortality in early childhood [8]. Beyond these quantifiable benefits, educated women tend to exercise greater decision-making power within households and foster democratic participation [9–11]. This interconnectedness underscores that gender parity in education is not merely a human rights issue but a strategic imperative for achieving inclusive economic growth, improved health, and sustainable social development.

Addressing gender disparities in education assumes even greater significance in low- and middle-income countries (LMICs), where limited resources, fragile infrastructures, and entrenched social hierarchies exacerbate existing inequalities, hindering progress towards the broader goals of gender equality. In these settings, educational inequities not only reflect but also drive broader structural vulnerabilities, limiting the capacity of individuals and communities to break intergenerational cycles of hardship [12, 13]. While primary school enrollment has improved in many LMICs, significant gender gaps persist, particularly in secondary and tertiary education, disproportionately curtailing women’s socioeconomic mobility and civic engagement [14]. Socio-cultural norms frequently prioritize boys’ education [15], relegating girls to domestic responsibilities and early marriage, a practice that continues to affect a substantial number of girls in LMICs [16]. These practices not only interrupt their educational trajectories but also erode human capital and negatively impact national development and public health outcomes [17]. These factors create formidable barriers for women seeking to enter and succeed in higher education, especially in demanding medical fields, where extended training and professional development are essential [18].

The urgency of addressing educational inequities in LMICs is further compounded by socioeconomic vulnerabilities and infrastructural deficiencies that disproportionately affect girls and women. Economic hardship often exacerbates gender-biased household resource allocation, prioritizing boys for limited educational resources (e.g., school fees, materials, transportation) due to perceived greater potential for future financial returns [19]. Inadequate educational infrastructure, particularly the scarcity of safe and gender-sensitive learning environments, further compounds these challenges. The lack of essential amenities, such as secure sanitation facilities and menstrual hygiene resources [20], coupled with the risk of gender-based violence in and around educational institutions [21], negatively impacts girls’ motivation, attendance, and academic performance, creating significant obstacles to their progression into higher education [20]. Consequently, achieving gender equality in education within LMICs is not merely an aspirational goal; it constitutes a crucial intervention that can transform social structures, enhance human capital, and establish a foundation for more equitable and resilient health systems.

While these pervasive challenges significantly impede girls’ access to and completion of basic education, the pursuit of gender equality in education extends beyond mere access. Even when women overcome these initial barriers and attain higher education, persistent societal pressures related to marriage, childbirth, and familial responsibilities continue to significantly shape their career trajectories [22, 23]. From early education through postgraduate training and beyond, women encounter societal expectations and norms prioritizing family obligations over professional ambitions, leading to compromised career progression, interruptions, or even complete abandonment [24]. This phenomenon warrants scrutiny, reflecting deeply ingrained societal values undermining women’s professional development and limiting their contributions to the healthcare workforce [25]. Existing research underscores the difficulties women face in balancing familial expectations with career aspirations [26, 27]. This issue is particularly evident in the South Asian context. For example, in Pakistan, women constitute a significant majority (80%) of medical college enrollments. Yet, a substantial proportion (50%) of graduated female doctors either do not enter practice or leave the workforce shortly after starting, according to the Pakistan Medical Council (PMC) [28]. In Pakistan, this loss of trained female physicians is a major contributor to the country’s overall doctor shortage. This example highlights how societal pressures can prevent women from fully realizing their professional potential in medicine. This highlights the critical need for supportive structures and policies that enable women to pursue their professional goals in medicine without sacrificing personal fulfillment or succumbing to these intense societal pressures.

In the Nepalese context, the interplay of socio-cultural norms and economic constraints creates a complex landscape for women’s educational and professional development, particularly within the medical field [29]. Nepal, classified as a lower-middle-income country in 2020 with a gross national income (GNI) per capita of $1,090 [30], faces considerable challenges in delivering adequate healthcare to its population [31]. While Nepal has made progress in improving women’s status [32], persistent patriarchal values and traditional kinship structures continue to shape expectations that confine women’s roles to the domestic sphere [32, 33]. These expectations can limit schooling opportunities for girls [34] and contribute to poorer health outcomes, as evidenced by research linking lower educational attainment to adverse reproductive health outcomes [35]. In this context, women’s educational disenfranchisement in Nepal cannot be separated from broader questions of economic justice and cultural transformation—both of which are integral to fostering a healthier, more equitable society.

Building upon this understanding of the broader socio-cultural and economic context in Nepal, it is crucial to examine the specific challenges faced by women within the medical profession. While an increase in female enrollment in medical programs in Nepal, exemplified by the 61.56% enrollment of women in the academic year 2018/19 [36], indicates progress, quantitative gains alone are insufficient due to the ongoing underrepresentation of women in the Nepalese medical workforce despite this rise in female medical student enrollment. Currently, women represent only 25% of Nepal’s generalist medical practitioners’ workforce [37]. A “hidden curriculum” of tacit values, behavioral norms, and institutional biases persists, directing female trainees away from high-prestige specialties and leadership positions while reinforcing traditional caregiving roles [38]. These implicit forces shape students’ clinical competencies [39], attitudes, and professional identities [40], ultimately influencing the caliber and diversity of future healthcare delivery [38, 41]. These challenges are compounded by economic disparities [42, 43] and uneven enforcement of gender equality policies [44], leading to persistent gender-based discrimination and harassment within academic and clinical settings [45, 46]. Taken together, these findings call for attention to the urgent need for a more transformative approach—one that not only broadens female participation in medical education but dismantles the entrenched cultural, economic, and institutional impediments that still stand in the way of equitable professional attainment and meaningful healthcare contributions by women.

Building upon the established context of pervasive gender inequities in Nepal, this study explores the challenges female medical graduates face as they transition into the medical profession. Focusing on their lived experiences, this research examines the interplay of gendered expectations, implicit biases, and structural inequalities embedded within their educational and clinical environments. It aims to illuminate the subtle yet powerful forces that shape their professional identities and influence their future roles as healthcare providers. Specifically, this study addresses the following research question: How do female medical graduates in Nepal perceive, navigate, and respond to the “hidden curriculum” of gendered norms and biases throughout their medical education, and how do these experiences shape their specialty choices, career paths, and contributions to the medical workforce?

## Methods

### Study design

This study employed a qualitative methodology, explicitly utilizing a narrative inquiry approach [47]. This design was selected as it is well-suited for exploring the lived experiences and personal stories of individuals, aligning directly with the study’s central aim: to understand how final-year female medical students in Nepal perceive, interpret, navigate, and make meaning of the gendered ‘hidden curriculum’ throughout their education and as they anticipate their career trajectories. Ontologically, narrative inquiry in this context adheres to a constructivist paradigm [48], acknowledging that individuals’ realities are subjective, socially constructed, and deeply embedded within their specific socio-cultural context. Epistemologically, it recognizes that knowledge is co-constructed through the interaction between the researcher and participant, emerging from the interpretation of the narratives shared [49].

Adopting narrative inquiry facilitates an in-depth exploration of the temporal and contextual dimensions of the participants’ journeys through medical education [50]. This allows for the capturing of complex sequences of events, the personal meanings ascribed to pivotal moments such as encountering bias or making career decisions, and the ways participants story their past experiences, present realities, and future aspirations in relation to pervasive gender norms and institutional structures. To ensure methodological rigor and transparency in reporting, the conduct and reporting of this study adhere to the Consolidated Criteria for Reporting Qualitative Research (COREQ) guidelines [51].

### Sample and setting

The study was conducted at a tertiary institution located in Bharatpur, a major urban center in Nepal’s Chitwan district. This institution was selected due to its prominent role as a leading provider of tertiary education and its large cohorts of undergraduate medical students, providing a relevant context for exploring the experiences of female medical students in a competitive learning environment.

Participants were recruited using a combination of purposive [52] and convenience sampling strategies [53]. Specifically, purposive sampling was employed to identify individuals who met predefined inclusion criteria designed to capture information-rich perspectives pertinent to the research question. The recruitment then proceeded based on convenience among those eligible within the selected institution. To ensure participants possessed a comprehensive understanding of the clinical environment and its associated challenges, the following inclusion criteria were established: Medical students were eligible if they (1) were enrolled in the final or fifth year of their undergraduate medical degree; (2) had completed at least 80% of their required clinical rotations; and (3) identified as female. This selection strategy aimed to gather perspectives from students with substantial clinical exposure, enabling them to offer rich insights into the factors influencing career progression within the medical field. Focusing on final-year students allowed the study to capture the nuanced perspectives of those nearing the transition from medical education to professional practice. A final sample size of fifteen participants was deemed sufficient for the study’s narrative inquiry approach, guided by the aim of achieving code and thematic saturation through in-depth exploration, focusing on the richness and complexity of experiences rather than statistical generalizability [54].

### Data collection

Data for this study was gathered through individual semi-structured interviews conducted following the COREQ guidelines. Semi-structured interviews were deemed the most appropriate method for data collection because they provide a flexible framework for exploring participants’ experiences and perspectives in depth while also allowing for emergent themes and unexpected insights to be captured [55]. This approach allowed the interviewer to establish rapport with the participants, creating a comfortable and supportive environment for them to share their stories and reflections on their experiences in medical education. No prior relationship existed between the researchers and the participants.

Each interview was conducted at a time and location convenient for the participant, lasting approximately 60 min. The data collection phase spanned from June 2024 to September 2024. A semi-structured interview guide was developed to facilitate a focused yet open-ended exploration of the research topic (see Table 1). This guide ensured that key areas of inquiry were covered while allowing flexibility to probe deeper into emerging themes and individual experiences. Recognizing the importance of allowing participants to express themselves authentically, they were given the option to respond in either English or Nepali. While English is the primary language of instruction in medical education, the choice to use their native language allowed for a more nuanced and comfortable expression of their experiences, particularly when discussing sensitive topics related to gender and cultural norms.

The researcher actively engaged with the participants throughout the interviews, employing probing and follow-up questions to elicit detailed and insightful narratives. This interactive approach fostered a deeper understanding of the participants’ perspectives and allowed for clarification and elaboration on key themes. All interviews were audio-recorded with the participant’s explicit consent, ensuring accurate capture of their narratives and facilitating subsequent analysis.

**Table 1:** Interview questions

No.	Interview Questions
1	Can you describe your experiences as a female medical student in Nepal?
2	How have societal expectations and cultural norms influenced your decision to pursue a medical career?
3	Have you encountered any gender-related challenges or biases during your medical education and training? If so, can you provide specific examples?
4	How have your family and peers supported or hindered your pursuit of a medical career?
5	What are your career aspirations after graduation, and how have your experiences as a female medical student influenced these aspirations?
6	Do you perceive any differences in the opportunities and challenges faced by male and female medical students and professionals in Nepal?
7	How do you envision your future contributions to public health in Nepal, and how do you think your experiences as a female medical student will shape those contributions?
8	What advice would you give to younger women who are considering a career in medicine in Nepal?

### Data analysis

A thematic analysis approach was employed to analyze the qualitative data gathered from the semi-structured interviews. This analysis was structured by the six-phase framework developed by Braun and Clarke [56], providing a systematic and rigorous method for interpreting the rich narratives shared by the participants.

To maintain analytical consistency and mitigate potential researcher bias, the lead author (AG), an academic with expertise in both qualitative research methodologies and clinical education, moderated all interviews and directed the analytical process. Initially, all audio recordings of the interviews were transcribed verbatim. To ensure accuracy and cultural appropriateness, interviews conducted in Nepali were first translated into English by the lead author and subsequently validated by the second author, who possesses fluency in both Nepali and English.

The research team, comprised of academics with extensive experience in qualitative public health research, then engaged in a thorough immersion process, involving multiple readings of the transcribed interviews. This in-depth familiarization with the data facilitated a comprehensive understanding of the participants’ experiences and viewpoints. Subsequently, the team systematically identified and extracted significant textual segments from the transcripts, each representing distinct ideas, experiences, or reflections expressed by the participants. These significant segments were then synthesized to capture their core meaning while retaining their essential content.

An inductive approach to coding was utilized [57], whereby these synthesized segments were assigned descriptive codes derived directly from the data, rather than relying on preconceived categories. A comprehensive codebook was then created to organize these codes into meaningful categories based on shared conceptual attributes. The study aimed for code and thematic saturation, focusing on the depth and richness of themes rather than achieving data saturation [54]. A collaborative approach was implemented to ensure analytical rigor and consensus. Initial coding was performed independently by two researchers, and the codebook underwent iterative refinement through comparative analysis and team discussions. Any disagreements in coding or interpretation were resolved through team consensus meetings, where the relevant data extracts were reviewed, and a shared understanding was established. This iterative cycle of independent coding, comparative analysis, and discussion continued until the research team achieved consensus on the final set of themes and sub-themes.

This meticulous analytical process facilitated the consolidation of initial codes into overarching themes that captured the key patterns and insights present within the data. In the final stage of analysis, a detailed narrative account was developed to present the thematic findings, incorporating illustrative quotations from the participants to provide rich contextual evidence. To further enhance the trustworthiness of the analysis, a peer debriefing process was undertaken. An external expert in qualitative research reviewed the interview transcripts, the research methodology, and the emergent findings, providing valuable critical feedback and identifying potential biases or inconsistencies. The research team revisited the transcripts and audio recordings as needed to ensure the final report faithfully represented the participants’ voices. This comprehensive and collaborative approach to data analysis was designed to ensure the rigor, trustworthiness, and transparency of the findings, establishing a robust foundation for understanding the complex dynamics of gender within medical education and practice in Nepal. The entire data analysis procedure is summarized in Table 2.

**Table 2:** Data analysis process

Exemplar Meaning Unit (Quote)	Code	Category	Sub-theme	Theme
“My parents… believe a woman’s ultimate goal is marriage and motherhood. I feel caught between their dreams and my ambitions.”	Marriage	Societal Expectations	Family Pressure	The Marriage Mandate: Negotiating Family, Tradition, and Professional Aspirations
“The unspoken societal expectation is always there… It’s a constant reminder that my value as a woman is tied to my marital status.”	Norms	Societal Pressure	Internalized Norms	The Marriage Mandate: Negotiating Family, Tradition, and Professional Aspirations
“I refuse to let societal expectations dictate my future… I want to become a skilled doctor and make a difference.”	Defiance	Agency & Resistance	Defying Norms	The Marriage Mandate: Negotiating Family, Tradition, and Professional Aspirations
“When I expressed my interest in cardiology, some doctors suggested pediatrics or OB/GYN instead, saying those were ‘more suitable’ for women.”	Stereotypes	Gender Bias in Medicine	Specialty Stereotypes	The Gendered Clinic: Unveiling Bias in Medical Education and Practice
“In many of our textbooks… women’s health issues are often relegated to specific chapters or presented as variations of the male norm.”	Curriculum	Gender Bias in Curriculum	Lack of Inclusivity	The Gendered Clinic: Unveiling Bias in Medical Education and Practice
“I felt like I had to work twice as hard to prove myself. Some of the male surgeons would dismiss my questions or make condescending remarks.”	“BoysClub”	Gender Bias in Clinical Practice	“Boys’ Club” Mentality	The Gendered Clinic: Unveiling Bias in Medical Education and Practice
“My parents mortgaged our land… They often talk about how my future husband needs to be well-educated and financially secure.”	Expectation	Financial Burden	Family Expectations	Investing in Daughters, Expecting Returns: The Gendered Economics of Medical Education
“I feel a deep sense of obligation… It makes me question my desire to pursue a career in public health, which may not be as lucrative.”	Debt	“Daughter Debt”	Career Choices	Investing in Daughters, Expecting Returns: The Gendered Economics of Medical Education
“I want to break that cycle. I want to use my education to achieve my own goals and contribute to society.”	Empowerment	Agency & Empowerment	Challenging Norms	Investing in Daughters, Expecting Returns: The Gendered Economics of Medical Education
“Going abroad for further studies… It’s a chance to create my own path and define success on my own terms. It feels like a passport to freedom.”	Freedom	Transnational Mobility	Seeking Liberation	Transnational Aspirations: Negotiating Mobility, Marriage, and Medical Careers
“My family… want me to get married before I leave… It’s a delicate balancing act, trying to navigate my aspirations while respecting their wishes.”	Negotiation	Family & Cultural Norms	Negotiating Expectations	Transnational Aspirations: Negotiating Mobility, Marriage, and Medical Careers
“I believe that pursuing further studies abroad will… expose me to different healthcare systems… and bring those experiences back to Nepal.”	Impact	Global Perspective	Local Impact	Transnational Aspirations: Negotiating Mobility, Marriage, and Medical Careers
“I refuse to accept the status quo… I actively voice my opinions… and advocate for gender equality within the medical college.”	Challenge	Challenging Norms	Activism & Advocacy	Claiming Space: Agency, Resistance, and Redefining Success in the Medical Profession
“I want to be a skilled and compassionate doctor, but I also want to have a fulfilling life outside of medicine… I believe that achieving a balance is essential.”	Balance	Redefining Success	Work-Life Balance	Claiming Space: Agency, Resistance, and Redefining Success in the Medical Profession
“I actively mentor younger female students… By building a strong network… we can create a more inclusive and empowering environment.”	Solidarity	Mentorship & Solidarity	Supporting Each Other	Claiming Space: Agency, Resistance, and Redefining Success in the Medical Profession

### Rigor and reflexivity

To ensure the trustworthiness and rigor of the qualitative data and subsequent analysis, this study adhered to established criteria for qualitative research quality, encompassing credibility, dependability, confirmability, and transferability [58].

Credibility was established through a comprehensive member checking process. This involved actively seeking feedback from participants regarding the accuracy and interpretation of their narratives. Member checking was conducted in two phases. First, during each interview, the moderator (AG) summarized key points and emerging interpretations to allow participants to confirm or challenge their understanding. This ongoing member checking during data collection ensured that the researcher’s interpretations were aligned with the participants’ intended meanings. Second, after the initial thematic analysis, a summary of the key themes and supporting quotes was compiled and emailed to all 15 participants. They were invited to review the summary and provide feedback on the accuracy and resonance of the themes with their experiences. Participants were given two weeks to respond, and any feedback received was carefully considered and incorporated into the final analysis. This iterative process of member checking enhanced the credibility of the findings by ensuring that they accurately reflected the participants’ perspectives.

Dependability was achieved through a meticulous approach to data collection and analysis. The semi-structured interview guide was developed based on a comprehensive literature review and the author’s deep familiarity with the Nepalese context, particularly regarding gender disparities in education and career progression. Furthermore, experts in medical education and qualitative methodology reviewed the interview guide and research protocol to ensure their relevance and rigor. This thorough preparation and expert input contributed to the dependability of the data collection and analysis process.

Confirmability was ensured through transparent and detailed documentation of the analytical process. This included maintaining a detailed audit trail of the research process, including descriptions of how data were summarized, meaning units extracted, condensed, coded, and ultimately synthesized into categories and themes. This meticulous record-keeping allowed an independent auditor to trace the research process and verify the findings, enhancing the confirmability of the study.

Transferability, which pertains to the potential applicability of the findings to other contexts, was supported through detailed descriptions of the study participants, the research setting, and the data collection and analysis process. This comprehensive documentation allows readers to assess the relevance and transferability of the findings to their own settings and situations.

Reflexivity was addressed through the research team’s ongoing critical self-reflection. Recognizing that the researchers’ own positions—specifically, their roles as academics embedded within Nepalese education and health contexts, which provided an ‘insider’ perspective, and their distinct gender identities, with the lead author being male and the second author female—could potentially shape interpretations and introduce biases. Hence, to mitigate this, the researchers maintained reflexive journals throughout the study to document assumptions, preconceptions, and reactions to the data, facilitating critical examination of how their perspectives might influence the research process. Furthermore, the diverse gender composition of the core research team involved in data analysis is considered a strength, fostering a more balanced interpretive lens during discussions. Regular team meetings provided a crucial platform for open dialogue and reflexive discussions, where interpretations were rigorously challenged, compared, and iteratively refined to ensure emerging themes were demonstrably grounded in participant data rather than researchers’ preconceived notions. Additionally, the peer debriefing process with an external qualitative research expert, mentioned previously, served as another vital mechanism to identify and mitigate potential unconscious biases. This multi-faceted commitment to reflexivity aimed to enhance the trustworthiness and transparency of the research process, ensuring the findings authentically represented the participants’ experiences and perspectives.

### Ethical consideration

Ethical clearance was obtained from the Nepal Health Research Council (approval number– 437/2024) and the institutional review board of Chitwan Medical College. Written voluntary informed consent was obtained from each participant, who was assured of their right to withdraw from the study at any time.

## Results

### Participants characteristics

This study involved fifteen final-year female medical students from a tertiary institution in Bharatpur, Nepal, aged between 26 and 31 years. Nine participants had entered the medical program directly following high school, while three held prior undergraduate degrees in health sciences. All participants reported medicine as their first-choice career path, expressing a deep passion for healthcare. All fifteen participants reported being unmarried at the time of the interviews; marital status was not an inclusion criterion for the study. Table 3 provides a detailed overview of the participants’ socio-demographic characteristics. The key themes emerging from the analysis of these participants’ experiences are visually summarized in Fig. 1.

**Table 3:** Socio-demographic characteristics of participants

Pseudonym	Gender	Previous Education	Age	Medicine as First Choice
Anya	Female	High School	26	Yes
Maya	Female	High School	27	Yes
Nisha	Female	High School	27	Yes
Priya	Female	High School	26	Yes
Riya	Female	High School	26	Yes
Reena	Female	High School	26	Yes
Anjali	Female	High School	26	Yes
Bina	Female	High School	26	Yes
Chanda	Female	High School	26	Yes
Ishwari	Female	High School	26	Yes
Jyoti	Female	High School	26	Yes
Kalpana	Female	High School	26	Yes
Deepa	Female	Bachelor’s in Health Sciences	28	Yes
Gita	Female	Bachelor’s in Health Sciences	31	Yes
Hema	Female	Bachelor’s in Health Sciences	29	Yes

### Findings

**Fig. 1:**
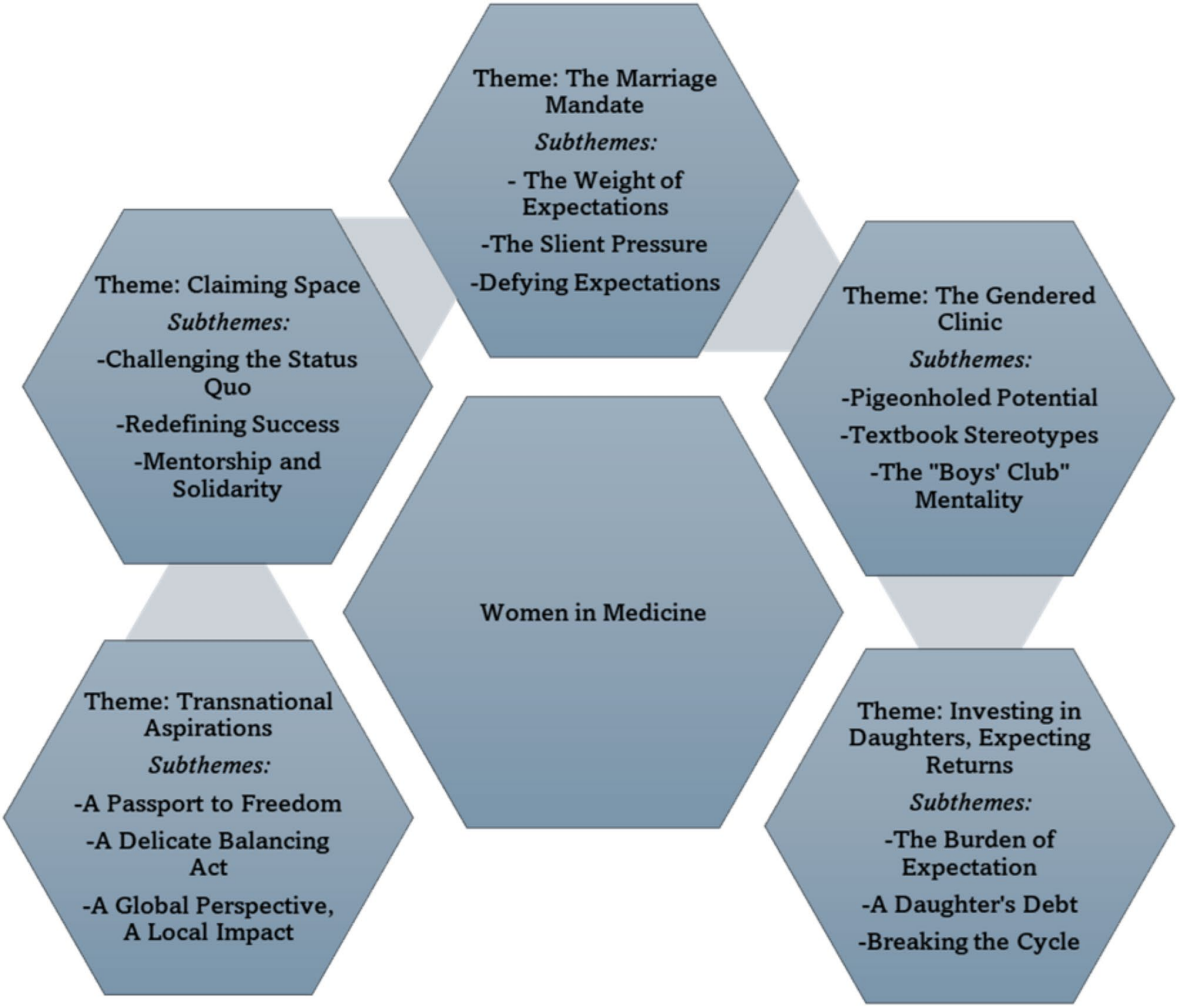
Key themes shaping the experiences of female medical students in Nepal

## Theme 1: the marriage mandate: negotiating family, tradition, and professional aspirations

This theme encapsulates the profound influence of societal expectations surrounding marriage and family on the professional aspirations of female medical graduates in Nepal. Participants described navigating a complex landscape where traditional gender roles often clash with their ambitions for a fulfilling medical career. This tension manifests in various ways, ranging from overt familial pressure to subtle societal cues, ultimately impacting their career choices, mental well-being, and sense of self.

### The weight of expectations

Many participants felt torn between fulfilling familial expectations and pursuing their professional ambitions. This internal conflict often stemmed from the deeply ingrained belief within their communities that a woman’s primary role is that of wife and mother.

My parents, like many others in our community, believe that a woman’s ultimate goal is marriage and motherhood. They supported my decision to study medicine, but I sometimes feel it’s seen as a temporary pursuit, something to do before I settle down and start a family. My mother often talks about how proud she’ll be when I’m married to a ‘good man’ and have children. It’s hard because I want to make them proud, but I also have this burning desire to become a skilled surgeon. I dream of specializing in neurosurgery, which requires years of further training and dedication. Sometimes, I worry that my family won’t understand or support that path, especially if it means delaying marriage or having children later in life. I feel caught between their hopes and dreams for me and my own ambitions. (Anya)

Anya’s poignant reflection illustrates the precarious position many female medical students occupy. While their families may support their initial pursuit of medical education, it is often perceived as a precursor to marriage rather than a lifelong career. The pressure to conform to traditional timelines for marriage and childbearing creates a significant burden, particularly for those aspiring to demanding specialties like neurosurgery.

### The silent pressure

Beyond explicit familial expectations, participants also described experiencing a more subtle yet pervasive form of societal pressure. This “silent pressure” is conveyed through unspoken cues, assumptions, and indirect messages that reinforce the primacy of marriage and family for women.

While no one explicitly tells me to get married and give up my career, the unspoken societal expectation is always there, hanging over my head like a cloud. It’s in the way relatives look at me with pity when I mention my plans for specialization, the questions neighbors ask about when I’ll find a husband, and the assumptions people make about my priorities. Even some of my female classmates, who initially shared my ambitions, are now talking about finding a husband and settling down. It’s a constant reminder that my value as a woman is often tied to my marital status, not my accomplishments as a doctor. It’s frustrating because I’ve worked so hard to get where I am, and I have so much more I want to achieve. (Maya)

Maya’s experience highlights the insidious nature of societal expectations. Even in the absence of direct pronouncements, the constant reminders and subtle cues from family, friends, and the wider community can create a sense of unease and pressure, impacting their self-perception and professional aspirations. This internalized pressure can be particularly damaging, leading to feelings of anxiety, stress, and self-doubt.

### Defying expectations

Despite these considerable pressures, some participants demonstrated a strong determination to challenge societal norms and pursue their professional goals. These women actively resisted the expectation to prioritize marriage and family, choosing instead to forge their own paths.

I refuse to let societal expectations dictate my future. I’ve seen how women in my family have sacrificed their dreams to fulfill their roles as wives and mothers. I respect their choices, but I want something different for myself. I want to become a skilled doctor, contribute to my community, and make a difference. I’m particularly interested in public health and working to improve maternal and child health in rural areas. I know it won’t be easy, and my family is already pressuring me to get married before I go abroad for further studies. But I’m determined to pursue my dreams, regardless of the challenges and obstacles I may face. By achieving my goals, I can inspire other young women to do the same. (Nisha)

Nisha’s narrative exemplifies the resilience and agency of some female medical students. Her determination to pursue her ambitions, even in the face of familial pressure, underscores a growing shift in attitudes among some women who are actively redefining traditional gender roles. Their pursuit of professional fulfillment inspires other young women and contributes to a gradual but significant change in societal norms.

## Theme 2: the gendered clinic: unveiling Bias in medical education and practice

This theme explores the pervasive gender biases that manifest within medical education and clinical practice, creating a challenging environment for female medical graduates. These biases, operating at both overt and subtle levels, shape opportunities, influence career trajectories, and perpetuate inequalities within the medical profession. The participants’ narratives reveal how these biases are embedded in their training, from clinical rotations to curriculum content and the overall culture of certain specialties.

### Pigeonholed potential

Participants described experiencing subtle but persistent pressure to conform to gendered expectations regarding specialty choices. This “pigeonholing” often steered them away from high-prestige or traditionally male-dominated specialties and towards those perceived as more “suitable” for women.

During my rotations in cardiology, I was often assigned tasks like taking patient histories and assisting with basic procedures. While my male colleagues were given more opportunities to observe complex surgeries and engage in critical discussions with the consultant. When I expressed my interest in pursuing cardiology, some doctors suggested I consider pediatrics or obstetric/gynecology instead, saying those fields were ‘more suitable’ for women. It felt like my potential was being limited by their preconceived notions about what women should and shouldn’t do in medicine. (Priya)

Priya’s account vividly illustrates how implicit biases can limit women’s career options. The subtle channeling of female students towards certain specialties, often based on outdated stereotypes about women’s capabilities and preferences, restricts their professional development and perpetuates gender segregation within the medical field. This undermines individual aspirations and deprives the profession of diverse perspectives and talents.

### Textbook stereotypes

Beyond clinical experiences, participants also highlighted the presence of gender biases within the medical curriculum itself. The underrepresentation of female patients in case studies and the tendency to present women’s health as a deviation from the male norm contributes to a skewed understanding of health and disease.

In many of our textbooks, case studies and clinical examples predominantly feature male patients, even when discussing conditions that affect both genders. Women’s health issues are often relegated to specific chapters or presented as variations of the male norm. This reinforces the idea that male physiology is the standard, while female bodies are somehow ‘different’ or ‘exceptional.’ This not only perpetuates stereotypes but also limits our understanding of women’s unique health needs. (Riya)

Riya’s observation points to a critical issue: normalizing male physiology as the standard in medical education. This perpetuates harmful stereotypes and has significant implications for patient care. By neglecting the specificities of women’s health, medical professionals may be less equipped to diagnose and treat female patients effectively, potentially leading to disparities in healthcare outcomes.

### The “boys’ club” mentality

Participants also described encountering a “boys’ club” mentality, particularly within surgical specialties. This culture, characterized by exclusionary practices, condescending attitudes, and subtle or overt discrimination, creates a hostile environment for female medical students and doctors.

Surgery is still very much a ‘boys’ club.’ During my surgical rotation, I felt I had to work twice as hard to prove myself. Some of the male surgeons would dismiss my questions or make condescending remarks. I once overheard a senior surgeon say that women are ‘too emotional’ to be good surgeons. It’s disheartening because I know I have the skills and dedication, but I worry that these ingrained biases will hinder my progress. It’s exhausting constantly having to fight for recognition and respect. (Reena)

Reena’s experience highlights the significant challenges women face aspiring to careers in traditionally male-dominated specialties. The “boys’ club” mentality creates a hostile environment, requiring women to constantly prove their competence and resilience. This not only creates undue stress and hinders their professional development but also discourages many talented women from pursuing these fields, further perpetuating gender disparities at higher levels of the medical profession.

## Theme 3: investing in daughters, expecting returns: the gendered economics of medical education

This theme explores the complex economic dimensions of female medical education in Nepal, highlighting how familial investment is often intertwined with expectations of social and economic returns, particularly related to marriage and future family support. This creates a unique set of pressures for female medical students, who must navigate their academic and professional pursuits and the weight of familial financial sacrifices and the associated expectations.

### The burden of expectation

For many participants, the financial investment in their medical education represents a significant sacrifice for their families, often involving mortgaging land or taking on substantial debt. While this investment is intended to provide opportunities, it also creates a burden of expectation, with families often anticipating specific social and economic returns.

My parents mortgaged our land and took out loans to pay for my medical education. They have poured all their resources into me, hoping I will become a successful doctor and uplift our family. While I am grateful for their support, I feel immense pressure to fulfill their expectations. They often talk about how my future husband needs to be well-educated and financially secure, someone who can ‘take care’ of me. It’s as if my education is not just an investment in my future but also a means to secure a good marriage and financial stability for the family. (Anjali)

Anjali’s poignant narrative reveals the complex dynamics at play. While families invest in their daughters’ education with hopes for a better future, this investment is often linked to traditional expectations of marriage and financial security. The pressure to find a “good match” and contribute to the family’s economic well-being can weigh heavily on female medical students, adding another layer of complexity to their already demanding academic pursuits.

### A daughter’s debt

The obligation to repay familial sacrifices financially and through fulfilling social expectations creates a feeling of “daughter debt” for many participants. This can lead to internal conflicts, particularly when their career aspirations diverge from familial expectations.

I feel a deep sense of obligation to my parents for the sacrifices they have made. They have invested everything in my education, hoping I will have a better life than they did. But sometimes, this feels like a debt I need to repay, not just with financial support but also by fulfilling their expectations of marriage and motherhood. It makes me question my desire to pursue a career in public health, which may not be as lucrative as private practice. I worry that my choices will disappoint them and that I won’t be able to ‘repay’ their investment in the way they expect. (Bina)

Bina’s reflection highlights the moral dilemma faced by many female medical students. The desire to pursue their passions, such as public health, can clash with the perceived need to choose a more financially rewarding career path to repay their families. This internal conflict can significantly impact their career choices and reinforce traditional gender roles.

### Breaking the cycle

Despite these pressures, some participants strongly desired to break the cycle of traditional expectations and redefine success on their own terms. They recognize the importance of financial independence and personal fulfillment as key components of their future.

I am grateful for my education and the opportunities it has given me. However, I also recognize that the traditional expectation for daughters to ‘marry well’ and prioritize family over career perpetuates a cycle of inequality. I want to break that cycle. I want to use my education to achieve my own goals and contribute to society. I believe that by pursuing my path and achieving financial independence, I can inspire other young women and show them they have the right to choose their future. (Chanda)

Chanda’s perspective reflects a growing awareness among female medical students of the need to challenge traditional norms. By prioritizing their goals and achieving financial independence, they aim to fulfill their aspirations and inspire other young women to break free from societal constraints and pursue their own paths. This represents a powerful shift towards greater gender equality and empowerment within the Nepalese context.

## Theme 4: transnational aspirations: negotiating mobility, marriage, and medical careers

For many participants, seeking opportunities abroad represents not only a chance for professional advancement but also an opportunity to navigate and, in some cases, escape the constraints of traditional gender roles and societal expectations within their home country. This pursuit of international experience creates a complex interplay between personal ambition, familial expectations, and a desire to contribute to improving healthcare in Nepal.

### A passport to freedom

For some participants, pursuing opportunities abroad offered a sense of liberation from the social and familial pressures they experienced in Nepal. This pursuit of international experience was seen as a way to prioritize their careers and define success on their own terms.

For me, going abroad for further studies isn’t just about advancing my medical career; it’s about gaining independence and escaping the suffocating expectations of my family and society. Here, I feel constantly pressured to get married, settle down, and prioritize family life. However, I can focus solely on my studies and career in the US [United States] without those pressures. It’s a chance to create my own path and define success on my own terms. (Deepa)

Deepa’s powerful statement encapsulates the desire for autonomy and self-determination that motivates some female medical graduates to seek opportunities abroad. The prospect of escaping the “suffocating expectations” of their home environment allows them to prioritize their professional development and pursue their ambitions without the constant pressure to conform to traditional gender roles.

### A delicate balancing act

However, pursuing transnational aspirations is not without its challenges. Participants also described the complex negotiations they face with their families, who often have concerns about their well-being and adherence to cultural norms. This creates a delicate balancing act between personal desires and familial obligations.

My family supports my ambitions, but they also worry about me going abroad alone. They want me to get married before I leave, believing it will provide me with stability and support. I understand their concerns, but I also fear that getting married might limit my mobility and flexibility. It’s a delicate balancing act, navigating my personal and professional aspirations while respecting my family’s wishes. I’m hoping to find a way to pursue my dreams without sacrificing my relationships with my loved ones. (Gita)

Gita’s experience highlights the tension between pursuing personal ambitions and maintaining familial harmony. While families may support their daughters’ desire for professional growth, they often express concerns about their safety and well-being abroad, suggesting marriage as a means of ensuring stability. This creates a complex negotiation for female medical graduates, who must navigate these conflicting expectations while striving to achieve their professional goals.

### A global perspective, a local impact

Despite the allure of international opportunities, many participants expressed a strong commitment to returning to Nepal and contributing to the improvement of healthcare within their own communities. They viewed their international experiences as a means of gaining valuable knowledge and skills they could bring back to their home country.

I believe that pursuing further studies abroad will not only broaden my medical knowledge and skills but also expose me to different healthcare systems and approaches. I want to learn from the best in the world and bring those experiences back to Nepal. I envision myself working in rural communities, providing quality healthcare to underserved populations. My international experience will make me a more well-rounded and compassionate doctor, better equipped to address our country’s unique health challenges. (Hema)

Hema’s perspective underscores the potential for “brain gain” rather than “brain drain.” By gaining international experience and expertise, these female medical graduates can be crucial in strengthening the Nepalese healthcare system and addressing health disparities within their communities. Their transnational aspirations are thus not simply about personal advancement but also about contributing to the betterment of their home country.

## Theme 5: claiming space: agency, resistance, and redefining success in the medical profession

Participants demonstrated agency through various forms of resistance, from directly challenging discriminatory practices to redefining conventional notions of success and fostering supportive networks among women in medicine. This theme highlights their proactive efforts to create a more equitable and inclusive environment for themselves and future generations of female medical professionals.

### Challenging the status quo

Participants described various ways in which they actively challenged the prevailing status quo within their medical education and training. This included voicing their opinions, questioning outdated practices, and advocating for greater gender equality within their institutions.

I refuse to accept the status quo where women are expected to be passive and compliant. I actively voice my opinions in class, challenge outdated practices, and advocate for gender equality within the medical college. I’ve even initiated discussions with faculty about the need for more female representation in leadership positions and a more inclusive curriculum. It’s not always easy, and I sometimes face resistance, but I believe it’s important to speak up and create space for myself and other women in this field. (Ishwari)

Ishwari’s active involvement as a student representative demonstrates a powerful form of resistance. By directly challenging existing norms and advocating for institutional change, she and others like her are working to create a more inclusive and equitable environment for women in medicine. This proactive approach is crucial in dismantling systemic barriers and fostering a culture of gender equality.

### Redefining success

Participants also expressed a shift in their understanding of what constitutes success in the medical profession. They challenged the traditional emphasis on career advancement and external recognition, prioritizing instead a more holistic definition of success that encompasses personal well-being and work-life balance.

For a long time, I thought success meant becoming a renowned specialist, working long hours, and sacrificing personal life for my career. But now, I realize that success can be defined in many ways. I want to be a skilled and compassionate doctor, but I also want to have a fulfilling life outside of medicine. I want to have time for my family, pursue my hobbies, and contribute to my community. Achieving a balance between my personal and professional life is essential for my well-being and ultimately makes me a better doctor. (Jyoti)

Jyoti’s reflections highlight a significant shift in perspective. By prioritizing work-life balance and personal fulfillment, these women are redefining success on their own terms. This challenges the traditional, often male-centric, model of professional success that prioritizes career advancement above all else. This redefinition of success is crucial for creating a more sustainable and fulfilling career path for women in medicine.

### Mentorship and solidarity

Recognizing the challenges they face, participants emphasized the importance of creating supportive networks and fostering a sense of solidarity among women in medicine. Mentorship, both formal and informal, played a crucial role in empowering and supporting female medical students and graduates.

I believe it’s crucial for women in medicine to support each other and create a sense of community. I actively mentor younger female students, sharing my experiences and offering guidance. I encourage them to pursue their dreams, challenge stereotypes, and believe in their own abilities. I also connect them with female doctors who can serve as role models and provide further support. By building a strong network of female mentors and allies, we can create a more inclusive and empowering environment for women in medicine. (Kalpana)

Kalpana’s emphasis on mentorship and solidarity highlights the power of collective action. By creating supportive networks and sharing their experiences, women in medicine can empower each other, challenge discriminatory practices, and foster a greater sense of belonging within a traditionally male-dominated field. This sense of community is essential for promoting gender equality and creating a more inclusive and supportive environment for all.

## Discussion

The “marriage mandate,” as articulated by participants, constitutes a formidable social norm in Nepal, prioritizing women’s roles as wives and mothers, often at the expense of their professional ambitions. This finding aligns with broader patterns of gender inequality in education and employment observed across the Global South, where socio-cultural norms frequently impede women’s access to and advancement within higher education and professional spheres [59–61]. While Nepal has witnessed notable progress in the enrollment of female medical students [36], deeply entrenched gender disparities persist within the medical profession. Alarmingly, only 19% of specialist medical practitioners in Nepal are female [37] a stark statistic that underscores the enduring challenges women face in pursuing and sustaining medical careers. The narratives shared by participants illuminate the complex interplay between individual agency, familial dynamics, and pervasive societal pressures. While some participants expressed a resolute determination to defy these expectations and forge their own paths, they also poignantly described the emotional and, at times, financial burdens associated with challenging deeply ingrained traditional norms. This internal conflict reflects the struggles of women globally who grapple with reconciling personal aspirations with familial obligations and societal expectations [62]. Indeed, globally, women dedicate 3.2 times more time than men to unpaid care work—an average of 4 h and 25 min per day [63]. This disproportionate burden of unpaid care work significantly constrains women’s capacity to pursue demanding careers, particularly in fields like medicine, which demand extensive time commitments and unwavering dedication.

The pressure to marry early and prioritize family life has profound implications for women’s career trajectories, particularly within Nepal’s already strained healthcare system. 2021 data from the Government of Nepal, Ministry of Health and Population, reveals that only 57% of births in Nepal are attended by skilled health personnel, and maternal mortality rates remain alarmingly high, especially in rural areas [64]. The perpetuation of the “marriage mandate” can further exacerbate these critical challenges by curtailing the career paths of female doctors, leading to a critical shortage of skilled healthcare providers, particularly in underserved communities where their expertise is most needed. This resonates with findings from Raza et al. [28] in Pakistan, who identified that patriarchal societal structures, including prevalent stereotypes against working women and the prioritization of early marriage, significantly hinder women’s ability to practice medicine. Their research revealed severe work-life conflict among female doctors, who often find themselves caught between socially ingrained gender roles as homemakers and their professional careers [28]. This not only represents a substantial loss of valuable human capital but also perpetuates gender disparities within the medical profession, limiting women’s representation in leadership and decision-making positions and further hindering the development of gender-sensitive healthcare policies.

Therefore, the “marriage mandate” in Nepal constitutes a significant public health concern with far-reaching consequences. By restricting female doctors’ professional development and limiting their career trajectories, this social norm directly undermines national efforts to strengthen the healthcare system and improve critical health outcomes, particularly for women and children. Addressing this deeply rooted issue necessitates a multi-faceted and comprehensive approach. This includes not only challenging prevailing social norms through targeted community-based interventions that focus on shifting attitudes surrounding marriage timing and spousal expectations [65, 66] but also implementing national policies that actively incentivize women’s full participation in the health workforce [67]. This integrated strategy, combining social and policy-level interventions, can strengthen health systems, enhance overall population health outcomes, and promote gender equality within the healthcare sector. It is also crucial to acknowledge and address the intersectionality of gender with other social categories, such as caste, ethnicity, and socioeconomic status, as these intersecting identities can further compound the challenges faced by women in the medical profession [68]. By bringing attention to the “marriage mandate” and its detrimental impact on female medical graduates, this study contributes significantly to a growing body of literature that underscores the critical need to address gender inequality in both education and employment as a fundamental pathway to achieving the Sustainable Development Goals and promoting equitable and improved health outcomes for all [69, 70].

The pervasive gender bias embedded within medical education and clinical practice in Nepal reveals a potent “hidden curriculum” that subtly but systematically reinforces stereotypes and constrains the aspirations of female medical students. Participants’ experiences expose a system where gendered expectations and biases permeate multiple facets of their training, ranging from curriculum content and daily clinical interactions to specialty choices and long-term career pathways. These manifestations of gender bias mirror broader global patterns, where medical education and training frameworks have historically been structured around androcentric norms, effectively centering the male body and experience as the default [71, 72]. The participants’ accounts of being steered towards “female-friendly” specialties such as pediatrics and obstetrics while being implicitly or explicitly discouraged from pursuing surgery or cardiology powerfully illustrate how deeply ingrained gender stereotypes can shape professional trajectories. This channeling of female medical students into specific specialties, often based on outdated perceptions of what constitutes appropriate roles for women, not only reinforces traditional gender roles but also severely restricts their career options and potential for advancement [73]. Such biases have a demonstrably detrimental impact on women’s professional development, contributing significantly to their persistent underrepresentation in leadership positions within the medical field. Despite women comprising 67.2% of the global health workforce, they hold a mere 25% of senior leadership roles, according to 2021 data from the World Health Organization (WHO), highlighting the pervasive vertical segregation that continues to plague the health sector [74].

Participants’ observations regarding the lack of inclusivity within medical curricula further underscore the urgent need for systemic reform. The persistent use of textbooks and case studies that predominantly feature male patients, even when addressing conditions that affect both genders, perpetuates the problematic notion of male physiology as the standard, effectively rendering female bodies as deviations or exceptions. Recent research has demonstrated that gender-insensitive curricula can lead to significant diagnostic gaps, delays in treatment, and demonstrably poorer health outcomes for women [75].

Furthermore, the experiences shared by the participants highlight the “boys’ club” mentality that can prevail in certain specialties, creating a hostile environment for female medical students and doctors. The constant need to prove their competence, the frequent experience of dismissive comments and microaggressions, and the struggle to overcome ingrained biases can negatively impact their well-being and professional development. This resonates strongly with research conducted by Lyubarova et al. [76], which found that female physicians were more likely to experience gender discrimination and sexual harassment, contributing to burnout, dissatisfaction, and attrition from the profession. Such workplace cultures directly impede the achievement of gender-equitable progress in the health sector and limit the pipeline of women who can serve as role models and mentors to younger cohorts, thereby perpetuating a vicious cycle of underrepresentation [77]. The implications of these findings extend beyond the individual experiences of female medical students. When medical professionals are trained within a system that systematically reinforces stereotypes and biases, it inevitably affects their clinical decision-making processes. It contributes to demonstrable disparities in the quality of care provided to patients. For instance, research has consistently shown that women are significantly less likely to receive adequate pain management compared to men and are also more likely to be misdiagnosed with certain medical conditions [78].

Beyond the clinical sphere, the gendered economics of medical education play a significant role in perpetuating gender inequality. The participants’ descriptions of “investing in daughters, expecting returns” underscore how daughters’ medical training is frequently viewed as a family strategy that, while facilitating upward mobility, remains tethered to traditional gender norms. While families often make significant sacrifices to support their daughters’ medical education, these investments are often accompanied by expectations of social and economic returns, primarily through marriage and adherence to prescribed gender roles. These dynamic parallels broader patterns observed in the LMICs, where families perceive their daughters’ educational achievements to enhance their “marriageability,” reinforcing patriarchal structures that ultimately constrain women’s autonomy [79, 80].

The narratives shared by the participants illustrate the weight of these expectations. Many expressed a profound sense of obligation to fulfill their families’ hopes, feeling pressured to prioritize marriage and family life over their professional aspirations. Such pressures can manifest as “daughter debt,” a form of moral indebtedness that compels young women to align their career choices with familial and societal demands rather than their personal interests. While increased female enrollment in higher education can offer new professional avenues, entrenched gender norms frequently prevent women from translating educational attainment into long-term career advancement [22]. In the Nepalese context, where many families grapple with limited resources, substantial investments in a daughter’s training, often including loans or mortgaging land, intensify the expectation of reciprocal returns. This scenario creates a dual bind: women face not only the societal imperative to marry and stabilize family honor but also the economic imperative to justify the family’s financial sacrifices. Such constraints narrow their professional horizons, compelling them to choose " appropriate " pathways for women or forego certain specialties altogether. This phenomenon is not unique to Nepal as research in both LMICs and high-income countries (HICs) indicates that women often shoulder unequal caregiving burdens and experience career interruptions that hinder their advancement in science and medicine [81, 82]. Thus, while contextual factors differ, the underlying pattern—where economic considerations intersect with gendered social norms—remains distressingly common across diverse settings. Therefore, when women are denied the full scope of their professional aspirations due to familial or societal pressures, the healthcare system loses valuable human capital and expertise. A 2024 report from WHO underscores the importance of leveraging the entire talent pool of trained health professionals to improve access to quality care, reduce health inequities, and enhance health system resilience [83].

While resilient health systems depend on numerous factors often beyond individual control, participants’ narratives demonstrated personal defiance against traditional expectations and a determination to forge their own paths within the medical profession. Their accounts resonate with feminist theories of agency and resistance, which emphasize the capacity of marginalized groups to contest and reshape social structures that perpetuate inequality [84]. The participants’ accounts of resisting the status quo, advocating for gender equality, and mentoring younger female students reflect a commitment to fostering a more inclusive and equitable medical environment. This collective endeavor aligns with global movements in academic medicine addressing entrenched gender disparities and seeking diversified leadership. Studies indicate that despite increased female participation in medicine, gender gaps persist in senior positions, research funding, and professional recognition [85, 86]. By rejecting restrictive frameworks that have historically defined professional advancement, these women align with a broader international community of female health professionals working to dismantle systemic biases and reshape institutional cultures.

Participants explored how transnational mobility can offer liberation and expanded opportunities but also necessitates careful negotiation of familial expectations, cultural norms, and personal desires. For some, pursuing further studies or medical practice abroad represents an escape from Nepal’s “marriage mandate” and societal pressures. This opportunity to focus solely on their careers, free from expectations of early marriage and family life, can be empowering. Research confirms that women who migrate for education and professional development often gain enhanced social capital, broader professional networks, and improved negotiating power within familial and community contexts [87, 88]. However, pursuing transnational opportunities presents complexities. Participants expressed concerns about navigating familial expectations and maintaining relationships while abroad. The desire to respect family wishes and maintain cultural ties creates a delicate balance as they reconcile personal and professional aspirations. This tension is compounded by persistent gendered norms that frame women’s mobility as contingent upon marriage or kinship, where female migrants’ trajectories are often shaped by normative assumptions regarding their roles in reproductive labor and caregiving [89, 90].

The pursuit of transnational medical careers is also influenced by structural factors, including global demand for healthcare professionals and unequal distribution of resources and opportunities between HICs and LMICs. The “brain drain” phenomenon, where highly skilled professionals migrate seeking better opportunities, can significantly impact public health in source countries like Nepal [91]. The outflow of health professionals from lower-resource settings exacerbates workforce shortages, undermines health system resilience, and delays progress toward achieving the Sustainable Development Goals (SDGs), including SDG 3 (Good Health and Well-Being) and SDG 10 (Reduced Inequalities) [92]. These global imbalances raise ethical questions about the responsibilities of medical professionals trained in low and middle-income countries and the need for policies that support sustainable healthcare workforce development [93]. However, while “brain drain” is often framed negatively, research on diaspora engagement and return migration suggests more nuanced outcomes. Studies document how returning health professionals bring valuable technical skills, global perspectives, and expanded professional networks that can enhance local healthcare systems [94, 95]. This transnational circulation of expertise can foster medical innovation, improve service quality, and address inequalities in healthcare access [96].

However, focusing solely on individual agency and internal networks has limitations. Without systemic reforms, these efforts risk reinforcing a model where women are expected to compensate for institutional shortcomings through personal perseverance and adaptability. Organizational interventions—including transparent promotion criteria, gender-sensitive policies, and structural accountability measures—are necessary to dismantle deeply embedded biases and create equitable career pathways [97, 98]. By advocating for broader policy reforms and institutional transformations, these women and their allies can shift the responsibility for change from individuals to the systems that shape professional opportunities, ensuring that their resilience, innovation, and leadership are met with tangible, enduring progress.

### Limitations and future research

While providing valuable insights into the gendered experiences of female medical students in Nepal, this study has several limitations that suggest important avenues for future research. The qualitative, single-institution design in a relatively urbanized region of Nepal limits the generalizability of findings to the country’s diverse educational and sociocultural landscapes. Future research should employ comparative studies across public and private institutions, rural and urban settings, and diverse linguistic, ethnic, and caste groups to capture a more comprehensive understanding of the challenges and aspirations of female medical students nationwide. Furthermore, the focus on final-year students provides a temporally limited perspective. Longitudinal studies tracking participants throughout their medical education and into their professional careers are needed to examine the evolving influence of the “hidden curriculum” and the long-term impact of institutional reforms. While this study acknowledged the intersectionality of gender with other social identities, future research should explicitly investigate the complex interplay of these intersecting identities (e.g., caste, socioeconomic status, ethnicity) and their influence on access to opportunities and career trajectories. Finally, future comparative research across different LMICs would determine whether the findings reflect Nepal-specific norms or broader global patterns of gender inequality in medical education. Future research should also explore the perspectives of male medical students and gender-nonconforming learners to provide a more nuanced understanding of gender dynamics in medical education. Investigating the perspectives of faculty, administrators, and policymakers could elucidate the institutional mechanisms that perpetuate gender inequalities and inform targeted interventions. Moreover, longitudinal studies tracking female doctors’ career progression and its impact on health system performance indicators would provide valuable insights. Finally, expanding the scope to include other health professions would offer a more holistic view of gender dynamics within the healthcare workforce.

## Conclusion and recommendations

### Conclusion

This study reveals the intricate barriers faced by female medical graduates in Nepal as they navigate entrenched patriarchal norms and systemic inequalities within their professional aspirations. Despite these constraints, their narratives demonstrate remarkable resilience and a determination to challenge traditional expectations, redefine success, and forge new professional identities. These findings resonate with global efforts to achieve gender parity in education and employment, emphasizing that equitable representation in the medical workforce is a strategic necessity for advancing public health and social justice.

### Recommendations

Based on these findings, we recommend the following actionable strategies to promote gender equity in the Nepalese medical field:

1. **Implement Gender-Transformative Medical Curricula**: Move beyond simply integrating women’s health content. Implement curricula that actively deconstruct gender stereotypes, address implicit bias, and promote critical reflection on gender dynamics in healthcare. This includes incorporating intersectional perspectives and training faculty in gender-sensitive pedagogy and assessment methods, focusing on actively changing mindsets.
2. **Establish Institutional Gender Equity Offices**: Create dedicated offices within medical institutions with the mandate and resources to monitor, investigate, and address gender bias and discrimination. These offices should be empowered to implement transparent policies, provide confidential reporting mechanisms, and conduct regular gender audits of institutional practices related to recruitment, promotion, and leadership.
3. **Develop Targeted Leadership and Mentorship Programs**: Implement structured leadership development programs specifically designed for female medical professionals. These programs should provide training in critical skills like negotiation and strategic planning, while also facilitating access to influential mentors and sponsors to accelerate women’s advancement into leadership roles.
4. **Mandate and Monitor Gender Quotas in Leadership**: To promote equitable representation in decision-making processes, it is essential to build upon Nepal’s constitutional commitments and existing principles of affirmative action. For example, the allocation of 33% of the budget for scholarships aimed at women [99] could be expanded to include a similar representation quota for women in all leadership and senior positions within medical colleges, hospitals, and health governance bodies. This structural intervention is crucial for accelerating gender parity and enriching perspectives within health governance.
5. **Implement Targeted Financial Support and Incentives**: To alleviate the financial burdens identified and incentivize service in underserved areas, establish targeted financial mechanisms for female medical graduates, including needs-based scholarships, tuition waivers, and debt relief or loan forgiveness programs conditional upon a commitment to practice in designated rural or high-need communities for a specified period. This addresses economic barriers and supports the distribution of the health workforce.
6. **Promote Family-Friendly Policies and Support Systems**: Implement and enforce policies that support work-life balance for all medical professionals, including flexible work arrangements, accessible childcare, and adequate parental leave. Couple these policies with initiatives challenging traditional gender roles in caregiving to create a more supportive environment for women in medicine.

These recommendations aim to shift from simply acknowledging inequities to actively transforming the structural and cultural conditions that perpetuate them. Empowering women within the medical profession will not only enrich the workforce but also enhance healthcare quality, responsiveness, and equity, contributing to a more just, healthy, and inclusive Nepalese society.

## Electronic supplementary material

Below is the link to the electronic supplementary material.

Supplementary Material 1

## Data Availability

The data supporting this study’s findings are available on request from the corresponding author. However, the data is not publicly available due to privacy or ethical restrictions.
